# Research hotspots and trends of acupoint and pain based on PubMed: a bibliometric analysis

**DOI:** 10.3389/fneur.2024.1498576

**Published:** 2025-01-17

**Authors:** Zhulin Wu, Wanjun Tan, Siyi Li, Weiqing Zhang, Mingbo Lai, Weijun Luo

**Affiliations:** ^1^Department of TCM, People’s Hospital of Longhua, Shenzhen, China; ^2^The Eighth Affiliated Hospital, Sun Yat-sen University, Shenzhen, China

**Keywords:** acupoints, pain, bibliometrics, acupuncture, transcutaneous electrical acupoint stimulation

## Abstract

**Objective:**

Acupoint-related interventions are the widely utilized modalities in traditional Chinese medicine for the alleviation of pain. This study aims to identify research hotspots and trends by conducting a bibliometric analysis of the relevant literature on acupoint and pain, thereby elucidating future research directions in this field.

**Method:**

A comprehensive search was conducted on PubMed for literature pertaining to acupoint and pain from January 2010 to August 2024. Subsequent bibliometric analyses, encompassing statistical evaluation of bibliographic data, keyword cluster analysis, and co-occurrence analysis, were conducted utilizing the Medpulse database and the Bibliometrix R-package.

**Results:**

A total of 742 articles from 179 journals were included in the analysis, with the majority focusing on complementary and alternative medicine or comprehensive research. The number of publications in this field has shown a consistent annual increase, involving contributions from 19 different countries of corresponding authors. China had the greatest contribution with 407 articles followed by Korea with 25 articles. The leading institutions in terms of publication volume are Chengdu University of Traditional Chinese Medicine, China Medical University, and Kyung Hee University. The topics covered in these articles include acupuncture, transcutaneous electrical acupoint stimulation (TEAS), randomized controlled trials, analgesia, zusanli (st36), systematic review, and anxiety, among others. The main cluster themes are intervention methods for various acupoints and the assessment of their efficacy.

**Conclusion:**

The bibliometric analysis has identified the intervention methods of acupoints and the evaluation of their efficacy in pain management as emerging research focal points. Additionally, anxiety is anticipated to emerge as a future research direction within this domain.

## 1 Introduction

Pain, the fifth vital sign (1), is the leading cause of disability globally, and the incidence of pain is gradually increasing due to the increase in pain-associated diseases (2). Moreover, the prevalence of pain endangers peoples’ physical and mental health and has generated a heavy global economic burden (3). Patients with all types of pain suffer from different degrees of pain, and pain has adverse effects on patients from the physiological, psychological, social, and spiritual aspects. Pain is not only a medical problem but also a social problem. Therefore, finding more measures to relieve pain is of clinical significance for improving the quality of life of patients. Currently, analgesics are still the main way to treat pain. Despite research supporting the safe use of analgesics, people remain reluctant to use these analgesics (4). In addition, deaths associated with opioids increased by another 35% by the end of 2020 (5). In the process of treatment with opioids, there are a series of problems, such as respiratory distress and the risk of side effects (e.g., constipation, nausea/vomiting, dizziness, sedation, and pruritus).

In recent years, the application of acupoints in pain treatment has become more and more widespread, which has attracted international attention. According to traditional Chinese medicine (TCM), acupoints are special sites on the body surface or under the skin along the meridians (6). Acupoint-related intervention is the application of TCM (including acupuncture, moxibustion, acupressure, and massage, etc.) to the acupoints to regulate qi as well as the blood of the human body (7). Furthermore, it is well known that pain could be relieved by the insertion and manipulation of acupuncture needles at acupoints (8). The physical stimulation of acupoints also demonstrated good clinical outcomes in pain treatment (9). A meta-analysis has indicated that transcutaneous electrical acupoint stimulation (TEAS) can reduce postoperative pain and the number of analgesics used after surgery (10). Thus, the comprehensive application of acupoints in pain is worth further investigation.

Bibliometric analysis is a method of analyzing articles through bibliometric theory and applying mathematical and statistical methods to analyze related articles (11). In addition, bibliometric analysis helps scholars discover‌ emerging areas and future directions of the research domain using visualization tools (12). By bibliometric analysis, this study systematically reviews the scientific publications correlated with acupoints and pain from 2010 to 2024 around the world, aiming to discover research trends, hotspots, and development trends in this field, so as to promote the application of acupoints in pain treatment.

## 2 Methods

### 2.1 Data sources

By using Medpulse,^1^ relevant scientific publications on the application of acupoints in pain were collected from the PubMed database. PubMed encompasses over 24 million citations pertaining to biomedical literature and facilitates daily access for millions of users. PubMed was selected due to its recognition as the principal resource among scholars in the medical domain and it remains the optimal tool in biomedical electronic research (13). Also, Mulpulse is an easy-to-use online tool with simple operation, clear process, and concise interface, and can be used to batch search research literature from PubMed using title, topic, publication type, or publication year. In this study, the search limited the topic (including title/abstract and keywords) to “acupoints” and “pain.” The scientific publications type is unlimited, the language is English, and the retrieval time was from January 1, 2010, to August 17, 2024. Inclusion criteria were articles associated with acupoints and pain, including clinical studies, basic research, animal studies, theory and methods articles, systematic reviews/meta-analyses, case studies, and clinical practice guidelines, etc. The exclusion criterion was publication before 2010 or after 2024.

### 2.2 Bibliometric analysis methodology

This study mainly used R package “bibliometrix”^2^ to complete bibliometric analysis, and the statistical analyses of journals, authors, keywords, institutions, countries, and co-occurrence networks in scientific publications were conducted. This study exported data from the database through the “PubMed export file” file format, and used the “bibliometrix” package in the R software (version 4.2.0) for data analysis. Data statistics were completed through “biblioAnalysis” and “summary” in “bibliometrix,” and then the graphical representation was performed using “Networkplot.” Finally, “Biblioshiny” was used for keyword analysis, co-occurrence network synthesis. All the above work was carried out on Medpulse. This bibliometric study did not require approval from an institutional review board or ethics committee.

## 3 Results

### 3.1 Annual publishing trends of acupoint and pain

**Figure 1:**
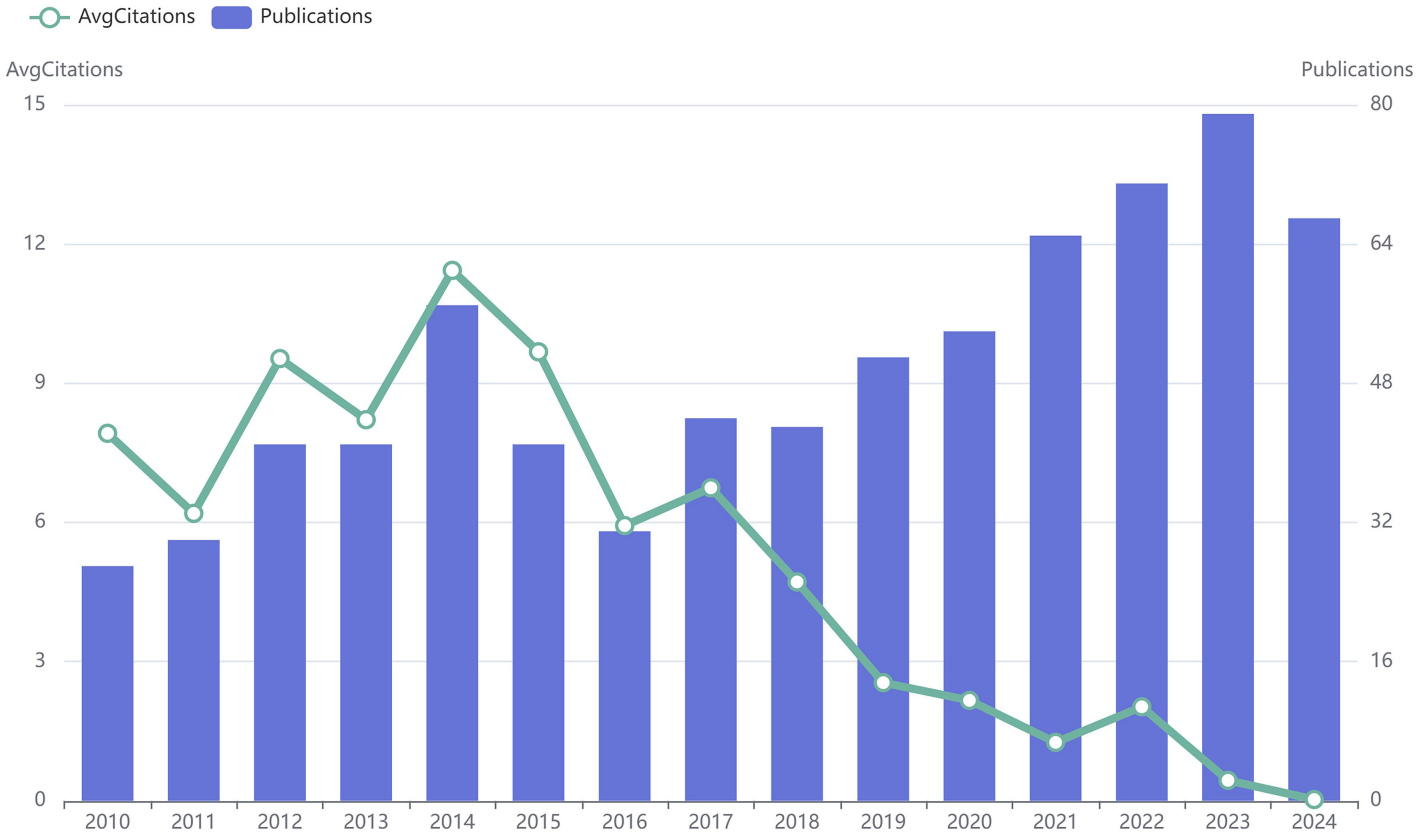
The annual number of publications and citations on “acupoints” and “pain” in the past 15 years. The blue bars represent the number of publications per year, and nodes indicate the number of citations.

From January 2010 to August 2024, there were 742 articles involving “acupoints” and “pain,” with an average of about 50 articles published every year. As shown in Figure 1, although the number of publications related to “acupoints” and “pain” declined slightly from 2014 to 2016, it has generally shown an upward trend in the past 15 years, which indicates that the application of acupoints in pain has gradually received more attention. The average annual citation frequency (average citations from all literature per year divided by the time span of year) has shown a decreasing trend after 2017, which may be related to the increase in the total number of publications.

### 3.2 Distribution of journals

**Figure 2:**
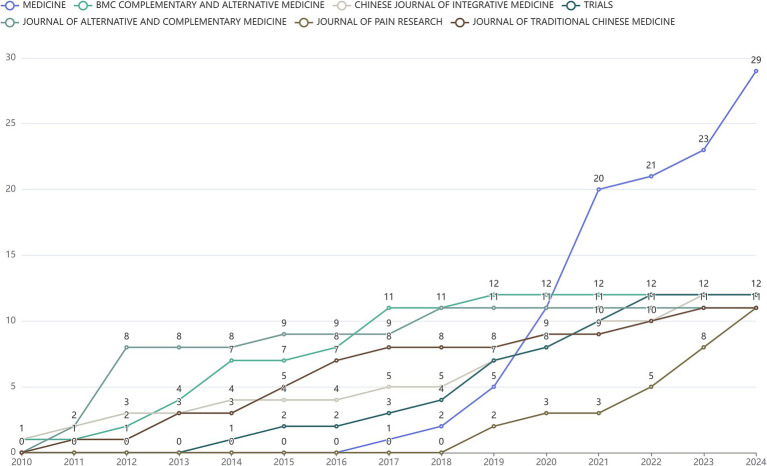
Line chart of publications by journal related to “acupoints” and “pain” in the past 15 years.

In the past 15 years, global publications on “acupoints” and “pain” have been published in 179 journals, and the frequency of the top 10 journals by year is shown in Figure 2. The publication frequency of the top 10 journals is increasing year by year. Among them, the journal with the largest number of publications was Medicine (22 articles), BMC Complementary and Alternative Medicine, Chinese Journal of Integrative Medicine and Trials tied at second spot with 12 publications each, and Journal of Alternative And Complementary Medicine was close behind (11 articles).

### 3.3 Distribution of authors and author productivity

**Figure 3:**
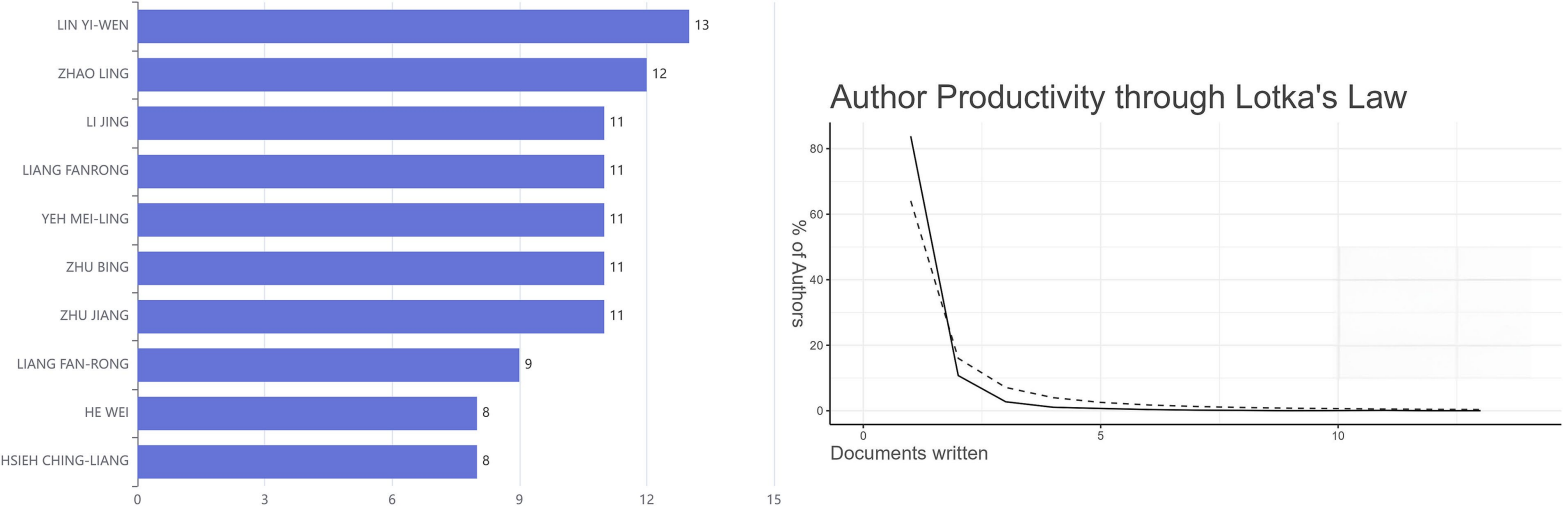
The number of publications by the top 10 authors from 2010 to 2024 (left) and the evaluation of author productivity through Lotka’s law (right). The solid line corresponded to the observed data, whereas the dotted line represented the theoretical relationship.

The relevant publications from January 2010 to August 2024 contained a total of 3,927 authors, and the top 10 authors with the most publications in this field were visualized (Figure 3). Among them, LIN YI-WEN published the most publications related to the research field, followed by ZHAO LING. Further analysis found that 5.795% of the 742 publications had multi-center collaboration. The productive authors have played a certain role in promoting the development of the research field of “acupoints” and “pain.” In addition, Lotka’s law indicated that authors who publish only one publication account for the majority: authors publishing a single publication have the highest frequency (0.838), and the theoretical value is 0.6407.

### 3.4 Distribution of institutions

**Figure 4:**
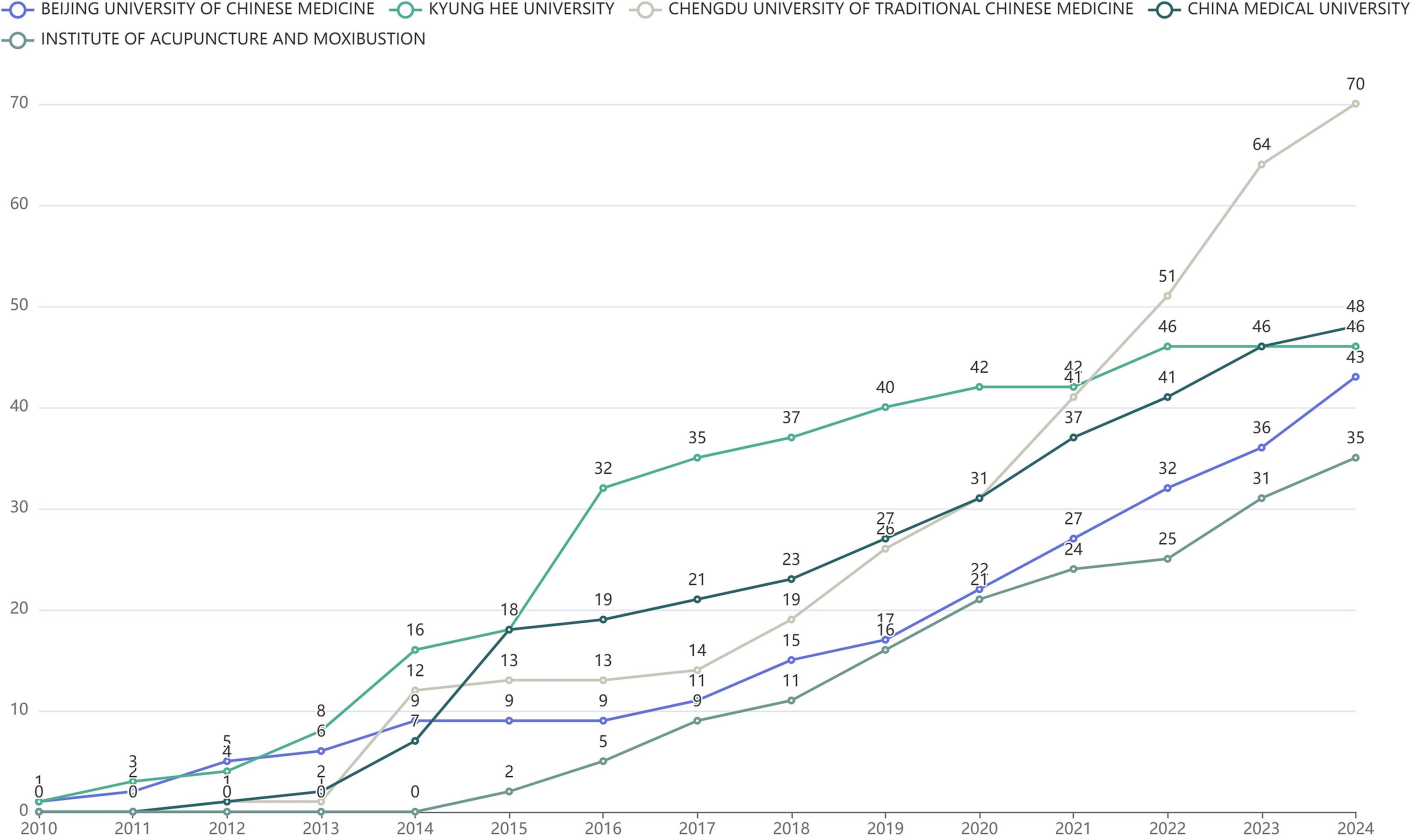
The number of papers published by institutions over time.

The statistical result of institutions that published publications in the field of “acupoints” and “pain” was displayed in Figure 4. Among all institutions, Chengdu University of Traditional Chinese Medicine (China) published the largest number of articles (frequency: 70), China Medical University (China) ranked second (frequency: 48), and Kyung Hee University (South Korea) ranked third (frequency: 46). Since 2010, the number of publications in this field by all institutions has steadily increased. In particular, the number of publications published by Chengdu University of Traditional Chinese Medicine increased to 51 in 2022 and has remained in the leading position.

### 3.5 Relationship between authors, institutions and sources (journals)

**Figure 5:**
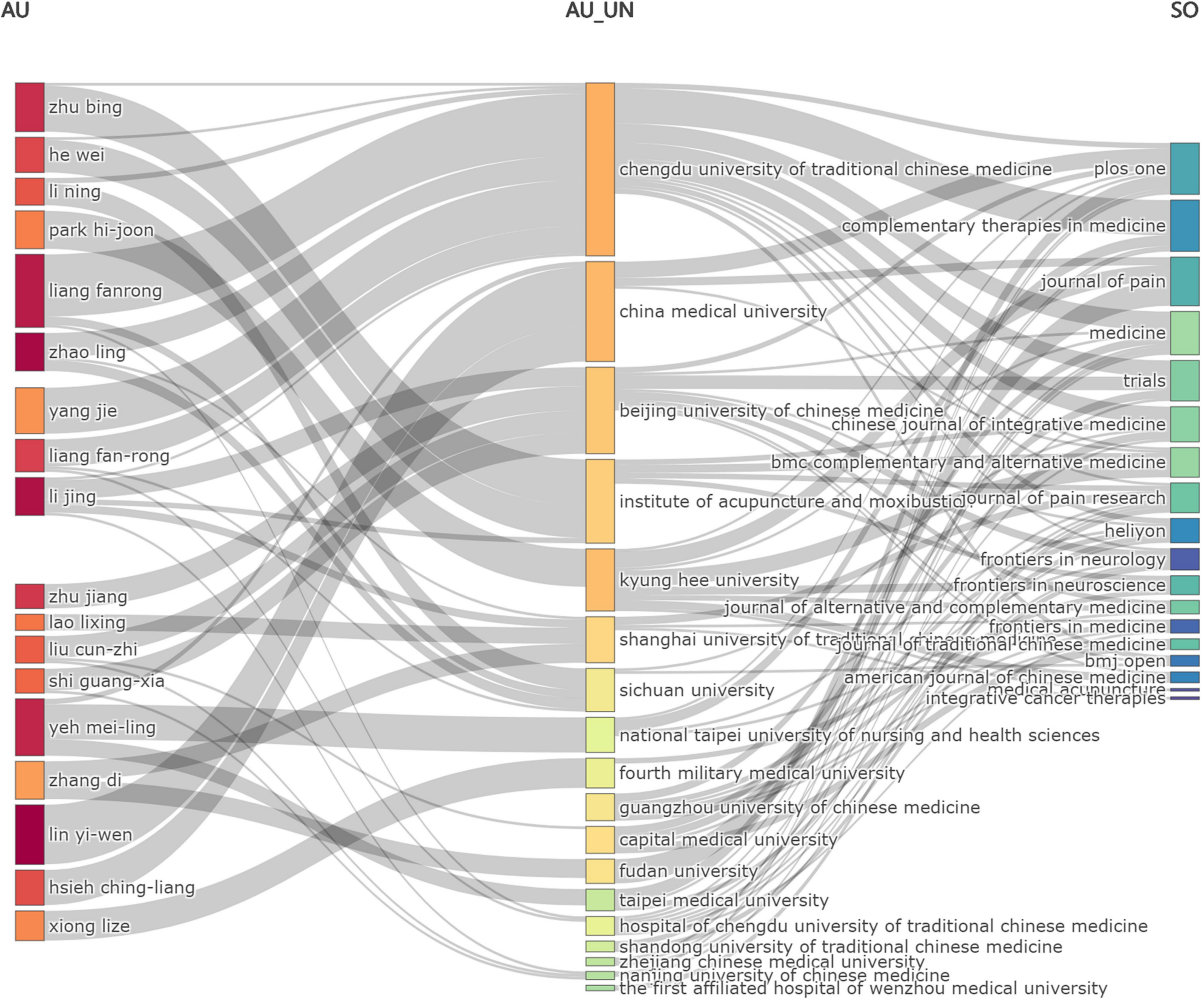
Sankey diagram of the publication related to “acupoints” and “pain” in the past 15 years. Sankey diagram showed the flow and relationship of the three fields: author (AU)-affiliated institution (AU_UN)-source (SO) for global publications. Different lines represented different diversion situations, and their length proportionally showed the number of publications contained in this branch.

The Sankey diagram was utilized to show the flow and relationship of the three fields: author-affiliated institution-source for global publications involving “acupoints” and “pain” from January 2010 to August 2024 (Figure 5). As shown in Figure 5, the author flowed most to Chengdu University of Traditional Chinese Medicine, indicating that this institution may have an important position in this field. Journals such as Plos One (comprehensive journal) and Complementary Therapies in Medicine have published articles collaborated by authors from different institutions, suggesting that scholars from different institutions have completed more comprehensive research through cooperation.

### 3.6 Distribution of authors’ countries or regions

**Figure 6:**
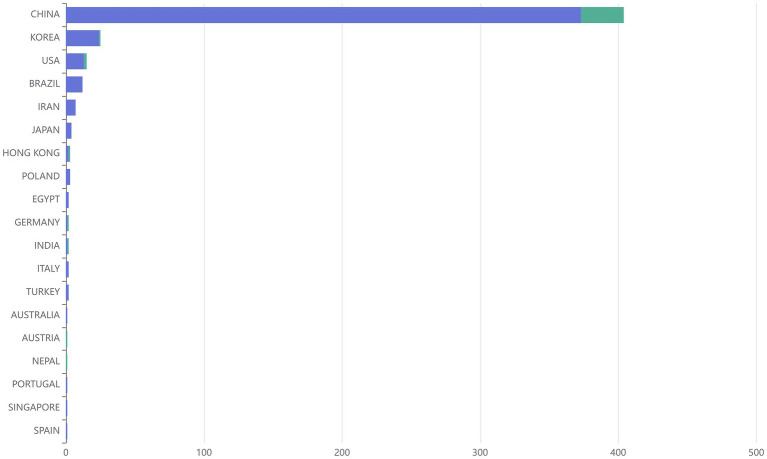
Bar chart showing the distribution of the top 10 countries of corresponding authors and the proportion of international cooperation.

The corresponding authors of publications correlated with “acupoints” and “pain” in the past 15 years have come from 19 countries. As shown in Figure 6, publications from a single country were displayed in blue, and publications from multiple countries were displayed in green; the country with the largest number of publications was China (including Hong Kong, the total number of publications was 407; the number of publications by multiple countries was 33; the number of publications by a single country was 374), South Korea ranked second, (the total number of publications by multiple countries was 1; the number of publications by a single country was 24), and the United States ranked third (the total number of publications by multiple countries was 2; the number of publications by single countries was 13). Besides, China and other Asian countries occupied the dominant position in this field. Further statistics on the distribution of authors’ countries or regions found that a total of 30 countries were involved, the top three rankings for author frequency were China (*n* = 1,221, 78.07%), South Korea (*n* = 102, 6.52%) and the United States (*n* = 57, 3.64%), and the most frequently cited articles originated from China (*n* = 1,320), South Korea (*n* = 242) and the United States (*n* = 82).

### 3.7 Analyses of word frequency and topic trends

**Figure 7:**
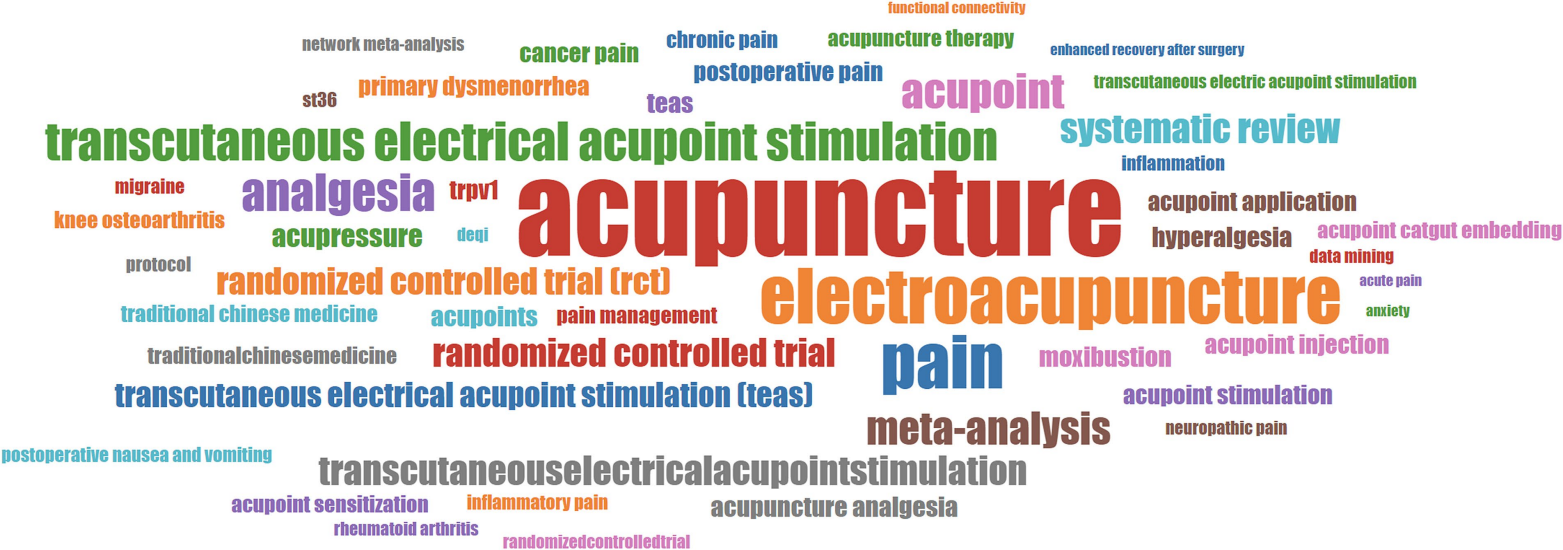
The top 50 keywords with the highest frequency of publications related to “acupoints” and “pain.” The greater the frequency, the larger the size of the keyword.

All the publications involved a total of 1,279 keywords, and these keywords were statistically analyzed to evaluate the different topics of the publications related to “acupoints” and “pain.” The top 50 keywords in terms of frequency were displayed by using the word cloud (Figure 7). After the same meaning words were merged, the keywords with high frequency are acupuncture (100), TEAS (*n* = 70), electroacupuncture (*n* = 42), pain (40), randomized controlled trial (RCT, *n* = 38), acupoint (*n* = 29), analgesia (*n* = 22), meta-analysis (*n* = 18), st36 (zusanli, *n* = 17) and systematic review (*n* = 15). Word frequency analysis suggests that intervention models at acupoints, including traditional acupuncture and TEAS, etc., are still the important focus, the form of research may be systematic reviews and RCTs, and the mechanism research mainly includes TRPV1 (transient receptor potential vanilloid subfamily 1) and inflammation-related pathways.

**Figure 8:**
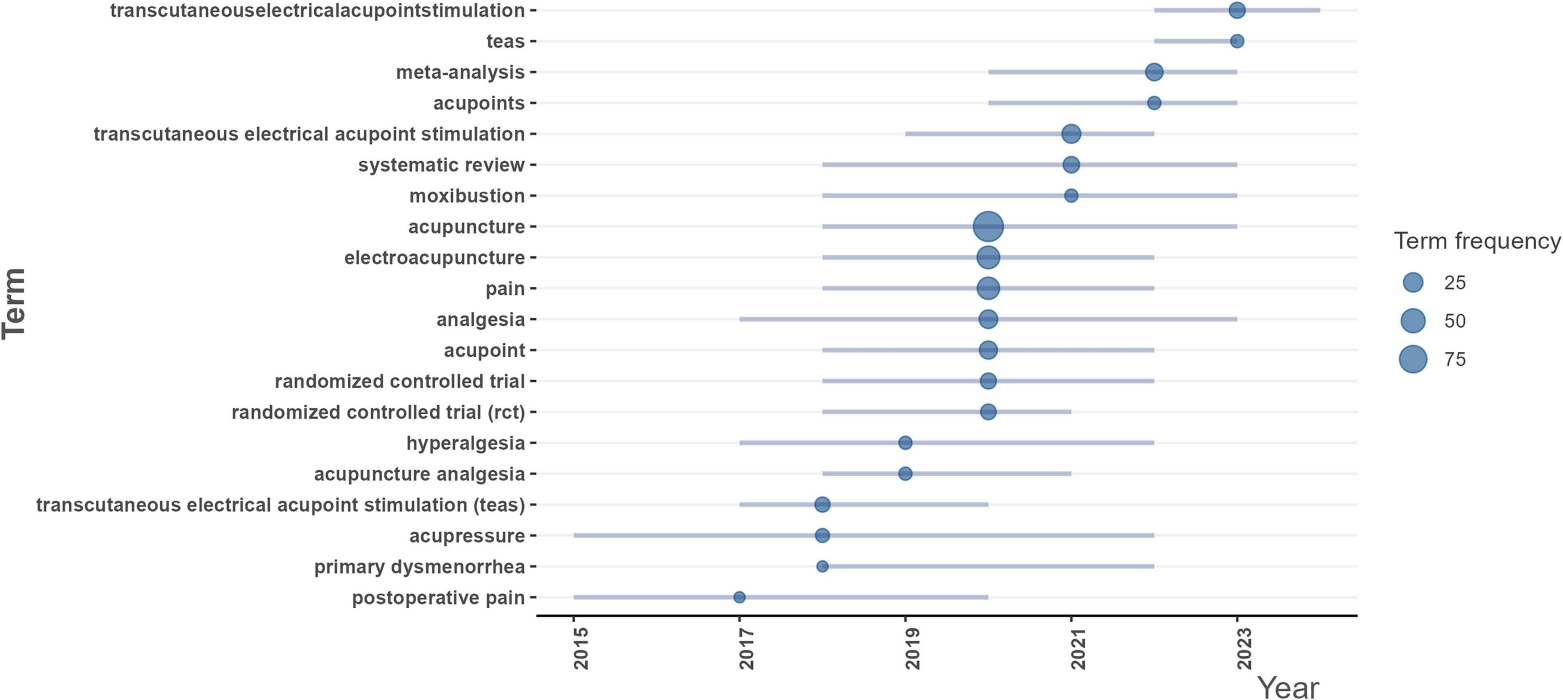
Research topic trends of publications related to “acupoints” and “pain.” Each line represented a keyword timeline, and the size of the bubble was proportional to the number of literature that used the keyword. The bubble is located at the midpoint of the timeline of the keyword.

Keywords analysis can potentially find the trending research topics both currently, and in the past. The analysis of topic trends suggests that TEAS and systematic review/meta-analysis continue to have a high frequency by 2023, indicating that they have always been the key research areas. Research associated with RCTs, analgesia, electroacupuncture, moxibustion, etc. has increased since 2018, with the median year being 2020, and this research is still active by 2023. Primary dysmenorrhea, hyperalgesia, postoperative pain, etc. are also important branches in this field (Figure 8).

### 3.8 Clustering by coupling

**Figure 9:**
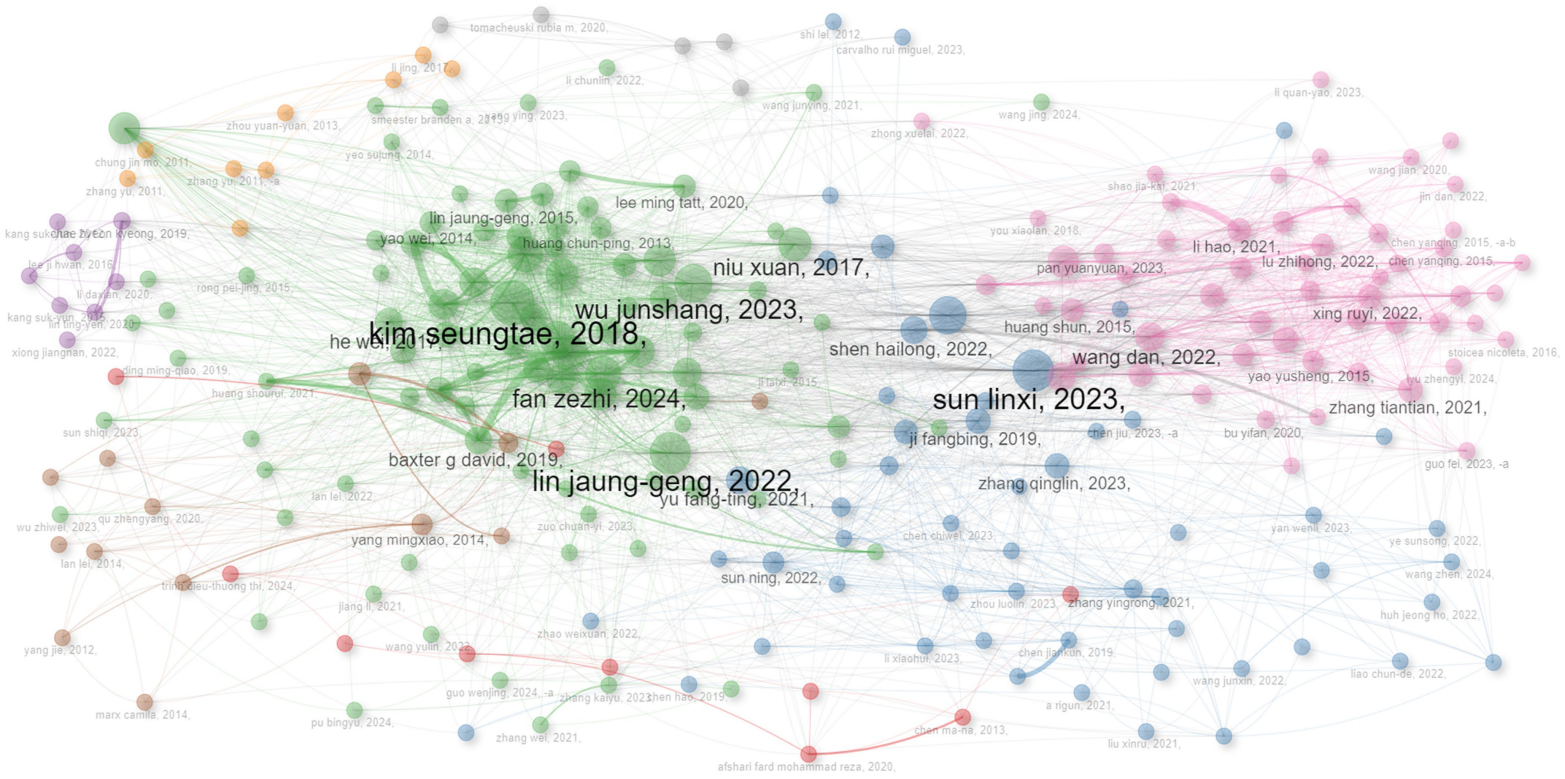
Clustering of publications related to “acupoints” and “pain” by coupling. Eight main cluster groups in this study were screened out, each cluster group showing different research characteristics. Among them, “acupuncture-electroacupuncture-analgesia,” “transcutaneous electrical acupoint stimulation,” and “acupuncture-acupuncture and moxibustion-analgesia” are the clusters with the highest centrality.

By analysis of clustering by coupling, this study screened out eight main cluster groups, each cluster group showing different research characteristics. Moreover, the three clusters with the highest centrality were further analyzed. Cluster one contained 99 publications, and this cluster was characterized by extremely high-frequency tags “acupuncture-conf 59.4%,” “electroacupuncture-conf 68.4%” and “analgesia-conf 71.4%”; the centrality of cluster one was 0.4718, indicating that this cluster occupied a relatively central position in the entire network. Cluster two included 61 publications, characterized by the tags “TEAS-conf 100%/87.5%” and “teas-conf 100%,” which also had strong network centrality (centrality: 0.4458). Cluster three, which contained 43 publications, was composed of “acupuncture-conf 3.1%,” “acupuncture and moxibustion-conf 100%” and “analgesia-conf 7.1%.” Although the centrality (0.333) of cluster three was relatively low, it still represented its importance in the network considering the size of the cluster (Figure 9).

### 3.9 Analyses of co-occurrence network and thematic evolution

**Figure 10:**
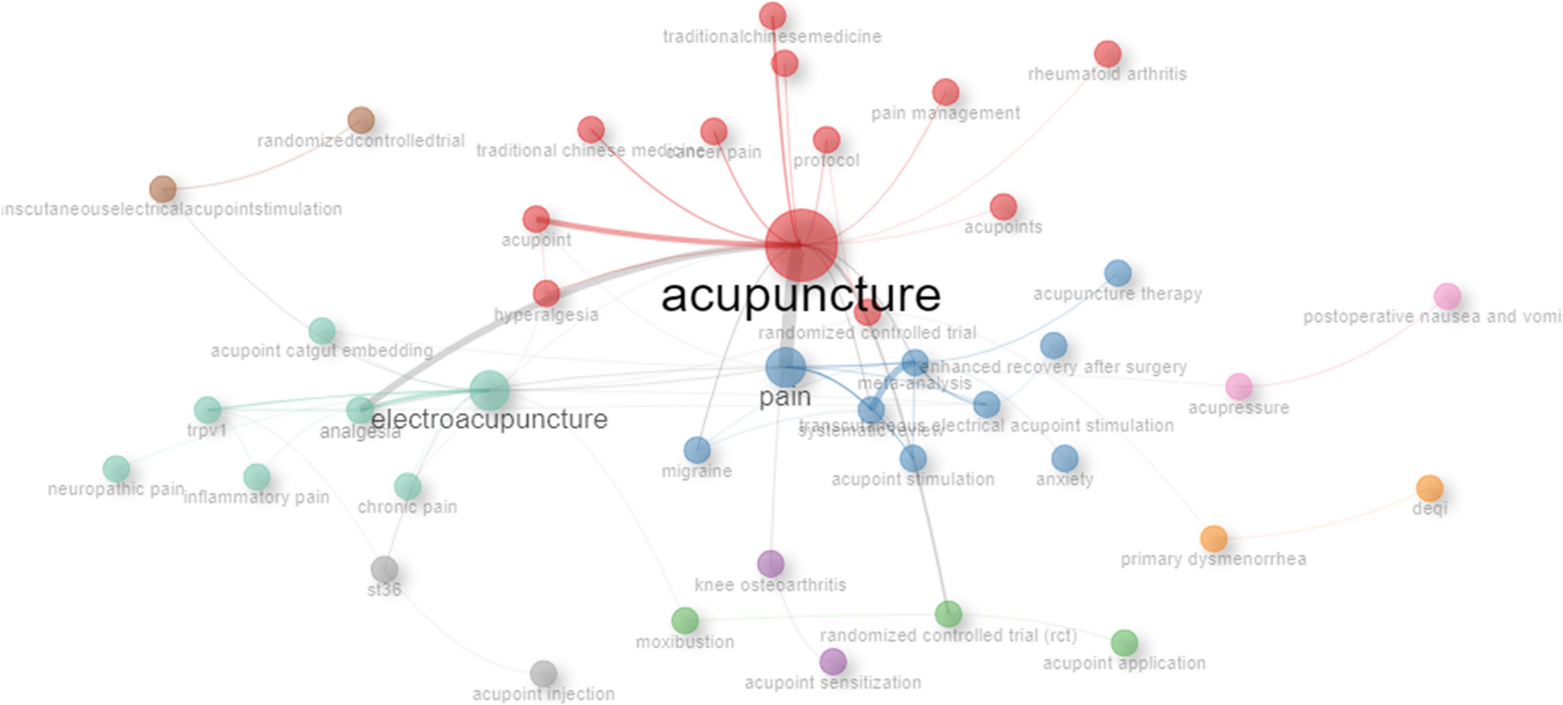
Keyword co-occurrence network of keywords in publications correlated with “acupoints” and “pain.” Nodes represented keywords, the node’s size was proportional to the frequency of keyword occurrence, and connections represented co-occurrence relationships or citation relationships between keywords.

The co-occurrence network analysis was performed on keywords in the publications related to “acupoints” and “pain,” and the results were as shown in Figure 10. In the co-occurrence network, nodes represented keywords, and connections represented co-occurrence relationships or citation relationships between keywords. “Acupuncture” was the most important node in the co-occurrence network, with the highest intermediary centrality (Betweennes = 414.1393), PageRank value (0.169), and closeness centrality (0.0137), indicating that “acupuncture” was a bridge connecting different research topics. “Pain,” “electroacupuncture,” “meta-analysis,” “RCT,” “analgesia,” and “TEAS” were also important nodes and played an important transmission role in the network. The network diagram also reflected the connection between keywords and the current hot spots in the field.

**Figure 11:**
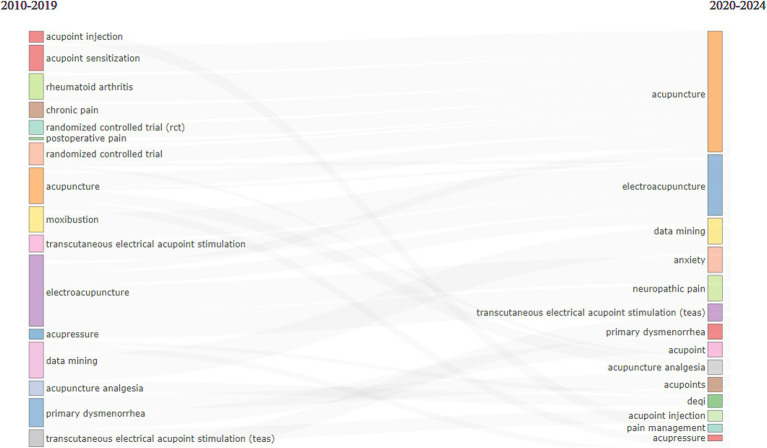
Thematic evolution in the fields of “acupoints” and “pain” at different time stages. Different lines represented different diversion situations, and their length proportionally showed the number of publications contained in this branch.

In terms of the evolution of research topics related to “acupoints” and “pain” in the past 15 years, acupuncture, electroacupuncture, TEAS, primary dysmenorrhea, data mining, acupoint injection, etc. have always been the topics of concern. After 2020, the increase in the research on anxiety and acupuncture reflected the dynamic development of this field over time and with the change of external factors (Figure 11).

## 4 Discussion

Bibliometric analysis is a methodological approach that facilitates both quantitative and qualitative analysis of literature and has been widely used in the medical field (15). Bibliometrics can not only be used to analyze the countries, institutions, authors, and journals of publications and their contributions to a certain research field but also provide a way to objectively measure the hot spots and trends in the research field (16, 17). As of now, bibliometric analysis of “acupoints” and “pain” has not been reported. In this study, the bibliometric method combined with visualization methods were used to analyze the literature related to “acupoints” and “pain.”

The annual trend analysis of publications indicates an upward trajectory in the number of English-language publications pertaining to the fields of “acupoints” and “pain” is on the rise, but the average citation frequency of the publications has a gradual downward trend, which may be related to the increase in the number of publications and the changes in some research directions. According to the statistical data, the main research units and active authors in the field were mainly from China and East Asian countries, and their high frequency of publications made them have greater influence and activity in this field. The authors of the publications in this study were primarily from specialized TCM institutions, and some authors were also from comprehensive universities, highlighting the interdisciplinary nature of the research. The authors and corresponding authors from China were in an absolutely core position in this field and have made important research contributions, which may be associated with the fact that the theory of acupoints originated in China. We suggest Chinese researchers engage in more multicenter clinical studies and collaborate with investigators from other disciplines. Korea, the United States, and Brazil also have a certain degree of activity in this field, indicating that the application of acupoints in pain has received international attention. The United States’ robust scientific research capabilities have led to significant achievements in acupuncture studies (18), and bibliometric data indicated that many publications on acupoints and pain were from the United States, suggesting that the United States may have more and higher-quality output in this field in the future. Korea, like China, has a long history of acupuncture and has included acupuncture in national legislation; acupuncture received legislative support from Brazil in 1988, and Brazil is the only country other than China to establish an acupuncture residency program (19). Research on acupoints and pain by scholars from different countries has advanced the systematization and spread of TCM, leading to its growing global recognition in recent years. In the future, we believe that more and more influential research will be published in this field by countries other than China. In addition, the journals with a large number of publications were mainly complementary and alternative medicine (TCM) journals or comprehensive journals. Figure 6 showed that there were many international collaborations in Chinese publications, indicating that international collaboration on acupoints and pain may be a future trend.

The word cloud can help users intuitively understand the keywords and hot spots. The keyword cloud map illustrates the most frequently occurring terms in the publications, with core words including acupuncture, TEAS, electroacupuncture, pain, RCT, acupoint, analgesia, meta-analysis, zusanli, and systematic review. Analysis of topic trends suggests that TEAS, and systematic review/meta-analysis have always been the key areas, and the activity of keywords such as RCTs, analgesia, electroacupuncture, and moxibustion demonstrated that intervention methods of acupoints and evaluation of their efficacy are the focus of research in this field. Furthermore, primary dysmenorrhea, hyperalgesia, and postoperative are also important topics of this field. Thus, evidence-based medicine, and intervention methods of acupoints for treating pain and pain types are still the future research directions.

Through the analysis of clustering by coupling, publications or authors can be systematically categorized based on the interactions among them. In this study, three main research clusters were extracted in the research of “acupoints” and “pain,” the centrality and influence of these clusters were also revealed in the research network. These clusters demonstrated that acupuncture, electroacupuncture, analgesia, and TEAS were the focus of research in this field from 2010 to 2024. A co-occurrence network is built based on the co-occurrence relationships of keywords in publications, which can identify the degree of relevance of each keyword in the network. The keyword co-occurrence network diagram showed the core position of keywords such as acupuncture, pain, and electroacupuncture in this field, indicating that the application methods of acupoints in pain are still topics of concern. Theme evolution is utilized to study the changes and development of key topics in specific research fields over time, which can help identify emerging trends, declining topics, and evolution paths of topics. Our data suggest that in addition to acupuncture, electroacupuncture, TEAS, dysmenorrhea, and data mining, pain-related anxiety and acupoint selection may be the future research directions.

In pain management, drug abuse and addiction have attracted increasing attention in recent years, and opioid overdose deaths and related issues are escalating, becoming a public health crisis in many countries (such as the United States) (20). The inadequacy of pain management strategies may be associated with a lack of comprehensive research on physiotherapy treatment or alternative treatment modalities, which are often recommended by clinical practice guidelines for the treatment of pain (21). Our research showed that conventional acupuncture, electroacupuncture, TEAS, moxibustion, acupoint injection, etc. were the main intervention methods for acupoints; zusanli was the key acupoint mentioned most frequently in the publications; the research methods are mainly RCTs and systematic reviews/meta-analyses, and TRPV1 and inflammation-related pathways are the most concerned in mechanism research. Acupuncture, which involves the action of inserting needles into acupoints, is a key part of TCM, and significant efforts have been made to prove that acupuncture has the characteristics of easy operation, low price, good efficacy, etc. (22, 23). According to the theory of TCM, acupuncture can activate qi and blood, improving the flow of qi, and acupuncture has been used widely to alleviate various pain conditions (24). TEAS is a non-invasive, safe, and comfortable treatment, that combines the effects of transcutaneous electrical nerve stimulation with acupoints stimulation, and previous research has shown that TEAS is effective in treating patients with various pain (25, 26). A multicenter randomized clinical trial showed that TEAS at combined acupoints before surgery was correlated with reduced chronic pain 6 months after surgery (27). A network meta-analysis displayed that TEAS may be a potential alternative for parturients as a simple and noninvasive intervention compared with epidural analgesia in terms of analgesic efficacy (28). It appears that TEAS can minimize the pain and infection risks associated with acupuncture, is more patient-friendly, and provides an innovative treatment option for pain. As technology advances, TEAS is expected to become an important tool in the field of pain in the future. However, TEAS-related clinical research is currently low (29) and high-quality multicenter trials are needed to broaden its clinical use. Besides, TRPV1 is found in both neural and non-neural cells at acupoints, and activating it might mimic acupuncture effects. Further research is needed to assess if TRPV1 acts as an acupoints-responding channel (30).

In TCM, zusanli (ST36) is an acupoint along the stomach meridian, which is located below the knee and on the tibialis anterior muscle. Stimulation of zusanli also has a wide range of effects such as analgesic and antispasmodic effects (31). Electroacupuncture on zusanli could significantly reduce colon lesions and relieve physical pain of colitis rats (32). Traditional acupuncture and electroacupuncture are widely used in treating inflammatory pain, and the acupoint with the highest frequency of application is zusanli (33). In terms of cancer pain, the most commonly used acupoint was also zusanli according to data mining research (34). Therefore, zusanli is a key acupoint in pain intervention, which is consistent with bibliometric results. In recent years, RCTs and meta-analyses in the fields of “acupoints” and “pain” have gradually increased. The designs of these RCTs would have been more reasonable and the results would have been more powerful if placebo control groups were set up scientifically (35–37). A meta-analysis from South Korea showed that acupuncture stimulation at acupoints significantly reduced pain in patients with low back pain compared with the control group (38). Another systematic review indicated that acupuncture improved postoperative pain on the first day after surgery, reduced the use of opioids, and could be used as an auxiliary treatment for postoperative pain (39). However, the quality of the original literature of most systematic reviews/meta-analyses is poor, and the credibility of these results needs to be further improved. Clinically, pain management for the elderly and adolescents is challenging, and there is currently a lack of RCTs related to acupoints. It is suggested that further high-quality RCTs be conducted in this field. Furthermore, we found that primary dysmenorrhea has always attracted the attention of scholars, which may be related to the lack of specific drugs in modern medicine. Research on anxiety may also be a hot spot and trend in the future, which is related to today’s social background. Chronic pain and anxiety often occur together in the clinical setting, and pain-induced anxiety can worsen the pain, creating a negative feedback loop (40). Electroacupuncture, which involves the stimulation of acupoints, has been clinically validated as a safe and effective approach for managing pain-associated anxiety (14). A meta-analysis showed that one-third of youth with chronic pain met the criteria for anxiety disorder. This represents a major clinical comorbidity whose prevention and treatment should be important health care priorities for youth with chronic pain (41). Thus, well-tolerated and youth-friendly regimens are needed, and the application of acupoints in pain-associated anxiety for youth deserves further research in the future.

While the bibliometric method is effective, it has some limitations. This study was limited by using only PubMed, excluding other sources, and was restricted to literature published in English, thereby not entirely eliminating the possibility of publication bias. Besides, although PubMed is the leading international biomedical search engine, the number of medical journals from various countries remains limited, and other databases like Web of Science are also available for bibliometric studies. Such limitation is typical in bibliometric studies. Moreover, although we provide an overview of the production situation concerning scholars, journals, institutions, and countries, as well as an analysis of emerging trends based on topics, this bibliometric method may be challenging to gather all important information and fully explore the potential connections in these relationships. As visualization technology advances, we recommend gathering publications from diverse databases to improve this research (42). Besides, acupoints are part of TCM, with extensive literature available in Chinese literature databases, but influential international journals were lacking, and it is necessary to select the international search engine-PubMed to study the global research hotspots and trends of acupoint and pain.

To sum up, this study conducted a comprehensive bibliometric analysis of the number of publications, authors, journals, and the distribution of keywords within the field of “acupoints” and “pain” over the past 15 years, providing a summary of the current research status, identified key research hotspots, and suggested potential future research directions. The findings indicate that the application of acupoints in pain management has been receiving increasing attention, and the intervention methods and efficacy of acupoints are still issues that need further study. Additionally, pain-related anxiety emerges as a prospective avenue for future research in this domain.

## Data Availability

Publicly available datasets were analyzed in this study. The data for this study can be found in the Pubmed (https://pubmed.ncbi.nlm.nih.gov/) and Medpulse (https://www.medpulse.cn/). All relevant data supporting the findings of this study are available within the paper, further inquiries can be directed to the corresponding author.

## Footnotes

^1^ https://www.medpulse.cn/

^2^ https://www.bibliometrix.org/home
